# A clinician’s perspective on the new organ mesentery and non-vascular mesenteropathies

**DOI:** 10.3389/fphys.2024.1336908

**Published:** 2024-09-04

**Authors:** Monjur Ahmed

**Affiliations:** Division of Gastroenterology and Hepatology, Department of Medicine, Thomas Jefferson University Hospital, Philadelphia, United States

**Keywords:** mesentery, mesenteropathy, mesenteric adenitis, mesenteric panniculitis, mesenteric adiposity, mesenteric neoplasms

## Abstract

Mesentery was discovered as a new organ in 2017. It is a continuous membranous tissue from the duodenojejunal flexure to the anorectal junction. It has distinct anatomy, physiology, and disease states. Primary mesenteropathies include vascular and non-vascular diseases. Some of them are common, and some of them are rarely seen in clinical practice. Secondary mesenteropathies occur when infection or malignancy in another organ spreads to the mesentery. Each entity has specific diagnostic and treatment protocols. Increased awareness of different mesenteropathies and an understanding of their various presentations at different stages of life can help in early diagnosis and improved clinical outcomes.

## Introduction

The mesentery is a double-fold peritoneum containing blood vessels, lymphatic vessels, lymph nodes, nerves, loose connective tissue, and fat. It was first depicted as a contiguous organ in 1,508 by Leonardo da Vinci. In 1885, Sir Frederick Treves presented it as a fragmented structure between the small and large intestines. In 2017, Professor John Calvin Coffey at the University of Limerick in Ireland reclassified the mesentery as a single, substantive, and continuous organ, i.e., an independent body part performing a particular function (Coffey et al., 2020). The mesentery extends from the duodenojejunal flexure to the anorectal junction, attaching the intestine to the posterior abdominal wall. It contains two layers continuous with both parietal and visceral peritoneum. Embryologically, most intra-abdominal organs (liver, pancreas, spleen, and intestine) are either developed in or on the mesentery. As a result, these organs are connected to the mesentery in adults. The mesentery and its contents can get involved in different benign and malignant diseases. Mesenteric diseases can be classified as primary or secondary mesenteropathies. Diseases originating in the mesentery with or without involvement of other organs are called primary mesenteropathies. On the other hand, diseases arising in other organs with subsequent progression and involvement of the mesentery are called secondary mesenteropathies. Sometimes, it remains unclear whether the disease originated from the mesentery or outside; then, it would be labeled as indeterminant mesenteropathy.

The different primary and secondary mesenteropthies are mentioned in Table 1. The anatomical and functional aspects of mesentery, as well as non-vascular primary mesenteropathies, will be discussed in this article.

**TABLE 1:** List of primary and secondary mesenteropathies.

Primary mesenteropathies	Secondary mesenteropathies
A. Vascular: acute mesenteric ischemia, chronic mesenteric ischemia and colonic ischemia B. Non-vascular 1. Mesenteric defect and internal herniation 1. Inflammatory mesenteropathies: primary mesenteric adenitis, mesenteric panniculitis 2. Mesenteric fibrosis 3. Mesenteric adiposity 4. Mesenteric cysts 5. Mesenteric volvulus 6. Mesenteric hematoma 7. Mesenteric lymphangioma or Mesenteric lymphatic malformation 8. Intestinal malrotation 9. Heterotrophic mesenteric ossification 10. Neoplasms: primary mesenteric lymphoma, mesenteric desmoid tumor, leiomyosarcoma, primary mesenteric neuroendocrine tumor, and primary mesenteric gastrointestinal stromal tumors (GISTs)	1. Secondary mesenteric adenitis 2. Mesenteric fibrosis due to JI-NET 3. Lymphomatosis 4. Carcinomatosis: pancreas, stomach, colon, uterus, lungs, breast 5. Sarcomatosis 6. Infectious: tuberculosis, actinomycosis, hydatid cyst

### Anatomical aspects

The mesentery is a double layer of peritoneum that encloses the intestines and contains loose connective tissue, fat, blood vessels, lymph vessels, lymph nodes, and nerves. The mesothelium covers both sides of the mesentery, which is continuous, with the parietal peritoneum lining the abdominal and pelvic walls and the visceral peritoneum lining the internal organs (Federle and Raman, 2015). The whole mesentery spans like a fan from the duodenojejunal flexure to the rectum and remains attached to the posterior abdominal wall by Toldt’s fascia (Coffey et al., 2014). As it travels downwards towards the rectum, it acquires different names in different parts of the abdomen and pelvis. Mesentery of the small intestine is called mesentery proper, which attaches the jejunum and ileum to the posterior abdominal wall. The root of the mesentery extends downwards from the duodenojejunal flexure (ligament of Treitz) and to the right to the ileocecal region. It is narrow, about 15 cm long, and attached to the posterior abdominal wall. It is close to the hepatoduodenal ligament and the right side of the transverse mesocolon. The superior mesenteric artery, superior mesenteric vein, jejunal and ileal blood vessels, lymphatics, and nerves required to supply the jejunum and ileum are seen between the layers of the mesentery proper. The mesentery that holds the colon to the posterior abdominal wall is called mesocolon.

The right mesocolon attaches the ascending colon to the posterior abdominal wall vertically, extending from the right iliac fossa to the subhepatic region. It contains the right colic artery. The transverse mesocolon attaches the transverse colon to the upper part of the posterior abdominal wall horizontally, dividing the abdomen into a supramesocolic and an inframesocolic space. It contains the middle colic artery. The mesentery proper further divides the inframesocolic space into a small right and larger left inframesocolic area. The left mesocolon attaches the descending colon from the subsplenic space to the left iliac fossa. It contains the left colic artery. The mesosigmoid attaches the sigmoid colon to the posterior abdominal in the left iliac fossa in an inverted V-shaped pattern. It has sigmoid arteries. It is continuous with the left mesocolon above and mesorectum below. The mesorectum attaches the upper part of the rectum to the posterior abdominal wall, and it contains the superior rectal artery. The mesoappendix attaches the appendix to the posterior abdominal wall. It originates from the mesentery of the ileocecal region and includes the appendicular artery.

### Functional aspects

The mesentery performs a multitude of functions. Mechanically, as it holds the intestine to the posterior abdominal wall, it maintains conformation of the intestine and prevents the intestine from collapsing into the pelvis during walking, running, or when we take different postures (Coffey and O’Leary, 2016). The mesentery contains three major arteries (celiac artery, superior mesenteric artery, and inferior mesenteric artery), two major veins (superior mesenteric vein and inferior mesenteric vein), and their arcades and branches connected by arteriole, venules, and capillaries collectively called splanchnic circulation which supplies blood to and drains blood from the digestive organs (Rogers and Deshpande, 2010). The mesenteric lymphatic vessels transport lymph (collected by lymph capillaries from the interstitial space) from the intestine to the central venous system and help in interstitial fluid balance, immune response, and nutrient absorption (Aukland and Nicolaysen, 1981). Thus, the mesentery maintains lympho-vascular communication between the intestine and the rest of the body. Mesenteric lymph nodes (MLNs) are part of gut-associated lymphoid tissue (GALT). They can trap live intestinal bacteria and viruses, regulate the migration of T, B, dendritic, and natural killer cells to the intestinal mucosa, and play an essential role in immune hemostasis (Hammerschmidt et al., 2008). MLNs also respond to food allergens and play an important role in inducing tolerance to food proteins. MLNs are, in fact, the “first pass” organs for many microorganisms and nutrients in the lymphatic fluid transported from the intestinal mucosa (McCright et al., 2021). Mesenteric fat contains white adipose tissue and plays a significant role in maintaining energy homeostasis and intestinal physiological ecology by storing and releasing lipids according to metabolic and nutritional needs. However, excessive mesenteric fat can lead to metabolic disorders (Zhang et al., 2022). Mesenteric adipocytes can phagocytose bacteria translocated from the intestine and thus prevent systemic bacteria dissemination. Following contact with bacteria and bacterial products, they can also produce leptin, IL-10, and MCP-1 (monocyte chemoattractant protein-1)/human C–C motif ligand 2, which can control local intestinal inflammation and allow infiltration of immune cells into the mesenteric fat to enhance defense (Batra et al., 2012). Mesenteric adipocytes secrete CRP in response to local inflammation and bacterial translocation to mesenteric fat in patients with Crohn’s disease (Peyrin-Biroulet et al., 2012).

## Non-vascular mesenteropathies

### Mesenteric defects

It can be congenital or acquired. Acquired defects are usually due to trauma or surgery. Congenital transmesenteric defect is generally 2–3 cm in diameter and can cause internal hernia and bowel obstruction. Patients can present at any age with abdominal pain and bowel obstruction, which is not uncommon in the pediatric population but is extremely rare in the adult population, representing 0.2%–0.9% incidence of all small bowel obstructions in adults (Benyamini et al., 2016). It is challenging to diagnose this condition pre-operatively as the transition point of bowel obstruction can be identified in imaging studies like computerized tomography (CT) scans. Still, the mesenteric defect can be missed (Nouira et al., 2011). During surgery, the mesenteric defect is identified, the internal hernia is reduced, and the mesenteric defect is completely closed to prevent the recurrence of internal hernia and bowel obstruction. According to different case reports, the prognosis is good if surgery is done at the appropriate time. Mesenteric defect with an internal hernia should be considered in patients with unexplained small bowel obstruction.

### Inflammatory mesenteric diseases

There are two inflammatory conditions of the mesentery. These include mesenteric adenitis and mesenteric panniculitis.

Mesenteric adenitis is due to non-specific inflammation of a cluster of three or more mesenteric lymph nodes in the right lower quadrant of the abdomen (Macari et al., 2002). The exact incidence is unknown, but mesenteric adenitis has been found in up to 20% of patients who went to the operating room for appendectomy. The etiology includes viral (rotavirus, norovirus, HIV) or bacterial infection (*Yersinia* pseudotuberculosis and *Yersinia* enterocolitica, *salmonella*, *Escherichia coli*, and streptococci), inflammatory bowel disease and secondary to intra-abdominal inflammatory processes. Mesenteric adenitis can be primary or secondary. Primary mesenteric adenitis occurs without an identifiable intra-abdominal inflammatory process. In contrast, secondary mesenteric adenitis is due to the abdomen’s detectable inflammatory processes (appendicitis, diverticulitis, pancreatitis, inflammatory bowel disease, pelvic inflammatory disease). Terminal ileal infection is thought to be the major cause of primary mesenteric adenitis. Primary mesenteric adenitis is more common in children and adolescents up to the age of 15 and above the age of 64. Patients generally present with right lower quadrant (RLQ) pain mimicking acute appendicitis, fever, malaise, anorexia, and diarrhea. Mild RLQ tenderness is found on physical examination. Laboratory studies may show leucocytosis and elevated CRP. Ultrasound (considered the gold standard) or CT scan with contrast detects the presence of a cluster of three or more enlarged lymph nodes (8 mm or more in the short axis in at least one) in the right lower quadrant mesentery near the terminal ileum (Karmazyn et al., 2005). Primary mesenteric adenitis is self-limiting; the patient generally gets better within 4 weeks. Treatment is mainly supportive care with intravenous hydration and non-steroidal anti-inflammatory drugs (NSAID) (Otto and Nagalli, 2023).

Mesenteric panniculitis is an idiopathic benign condition characterized by localized chronic non-specific inflammation and fibrosis of adipose tissue of mesentery proper (Green et al., 2018). It has different names depending on the histologic progression of the disease. In the beginning, there is a predominance of fatty degeneration and necrosis when it is called mesenteric lipodystrophy. In the next phase, there is a predominance of inflammation called mesenteric panniculitis. As the disease progresses, chronic inflammation leads to fibrosis when the term sclerosing mesenteritis (SM) or retractile mesenteritis (RM) is used (Emory et al., 1997). For practical purposes, mesenteric panniculitis is the most commonly used term and preferred terminology at present (Wagner et al., 2022). Mesenteric panniculitis is a rare disorder with an estimated prevalence of less than 1% in patients with abdominal CT done for various abdominal symptoms (Daskalogiannaki et al., 2000). It is twice as common in males than females (incidence being 3% vs. 1.5%). The usual age of presentation is the sixth and seventhth decades of life, and the incidence increases with age. Although mesenteric panniculitis is considered an immune-mediated inflammatory process, the exact etiology remains unknown. Mesenteric panniculitis has been associated with different medical conditions which include acute pancreatitis, abdominal trauma, surgery, chronic infections like histoplasmosis, tuberculosis, syphilis, and Whipple’s disease; paraneoplastic manifestations of lymphoma, neuroendocrine tumor, colon, renal and prostate cancers; autoimmune diseases, sarcoidosis, IgG4-related disease (IgG4-RD), and also fibrosclerotic disorders - Sjögren’s syndrome, Riedel thyroiditis, retroperitoneal fibrosis, sclerosing pancreatitis, and primary sclerosing cholangitis (Martin-Gorgojo et al., 2014; Burns and Bhavnagri, 2016; Sharma et al., 2017).

The clinical presentation of mesenteric panniculitis varies. Up to 40% of patients may remain completely asymptomatic. Vague abdominal pain with nausea is the most common presentation. The abdominal pain is usually mid-abdominal but can be anywhere in the abdomen or pelvis due to the mass-like effect of inflammation and potential involvement of the small bowel. Other symptoms include vomiting, early satiety, and alteration of bowel habits. Patients may also complain of fatigue, anorexia, and weight loss due to chronic inflammation. Sometimes, patients may present with small bowel obstruction or acute abdomen. A tender mass/fullness can be palpable in the abdomen’s center or left upper or lower quadrant (Alsuhaimi et al., 2022). Laboratory studies may show mild leucocytosis, elevated CRP, and erythrocyte sedimentation rate (ESR). Mesenteric panniculitis is usually diagnosed on contrast-enhanced CT imaging. It may show characteristic thickening, fat necrosis, and calcification of the mesentery. A hyperattenuated, inhomogeneous, well-defined solid fatty mass can be seen at the root of the mesentery.

Mesenteric vessels can be enveloped, but there should not be any evidence of invasion of adjacent displaced small bowel walls. Two specific signs are frequently seen. A “pseudocapsule,” i.e., a thin (<3 mm) band of hyperattenuating soft tissue encasing the solid mass and separating it from normal mesentery, can be seen in 50% of cases of mesenteric panniculitis. The hypoattenuated normal fat density surrounding the enveloped mesenteric vessels is preserved – a highly characteristic “halo sign” is seen in 75% of cases of mesenteric panniculitis (Coulier, 2011). Tissue diagnosis is not required routinely. In equivocal cases, tissue can be obtained by laparoscopy, exploratory laparotomy, or CT-guided aspiration (Sulbaran et al., 2023). Histopathology shows a layer of foamy macrophages replacing mesenteric fat and fatty degeneration in the phase of mesenteric lipodystrophy. The second phase of mesenteric panniculitis shows chronic inflammation with lymphocytic and plasma cell infiltration, fat necrosis, a few neutrophils, foreign-body giant cells, and foamy macrophages. The third phase of sclerosing mesenteritis reveals sclerosing fibrosis and calcification (Issa and Baydoun, 2009). In oncologic patients with suspected panniculitis, PET/CT should be considered to exclude malignant mesentery involvement (Zissin et al., 2006). Asymptomatic patients with mesenteric panniculitis do not require any treatment. A “Watch and wait” approach should be adopted. Mesenteric mass generally remains stable for many years or may even regress. Patients with persistent symptoms due to mesenteric panniculitis need anti-inflammatory treatment. Nyberg et al. studied a few patients with sclerosing mesenteritis and mesenteric panniculitis. They found prednisone (20–40 mg per day tapering over 8–12 weeks) as a first-line agent to be effective in 75% of cases (Nyberg et al., 2017). Steroid-dependent mesenteric panniculitis was treated successfully with colchicine in a case report (Iwanicki-Caron et al., 2006). Different other medications were used successfully to treat mesenteric panniculitis. These include azathioprine (Tytgat et al., 1980), cyclophosphamide (Bush et al., 1986), pentoxifylline (Kapsoritakis et al., 2008), tamoxifen (Venkataramani et al., 1997), progesterone (Mazure et al., 1998), thalidomide (Ginsburg and Ehrenpreis, 2002), infliximab (Rothlein et al., 2010), and ustekinumab (Byriel et al., 2022). Recently, Cortés et al. (2022) did a retrospective study and found that prednisone plus colchicine was as efficacious as prednisone plus tamoxifen for the initial and long-term treatment of mesenteric panniculitis. In a small open-label pilot study, three adults with symptomatic mesenteric panniculitis were given low-dose naltrexone (LDN) 4.5 mg nightly for 12 weeks. Their mesenteric panniculitis subjective score and physical, social, emotional, and functional wellbeing improved (Roginsky et al., 2015). This study was promising as LDN modulates the immune system and increases blood levels of enkephalins and endorphins. Currently, corticosteroids are considered the first line of therapy for symptomatic patients with mesenteric panniculitis, which should be tapered off after a few weeks. Steroid responders should be offered long-term maintenance therapy with thiopurines. Steroid non-responders should be tried with other therapy forms, including colchicine, tamoxifen, pentoxifylline, anti-TNF, thalidomide, and LDN. The role of surgery is limited only to complicated mesenteric panniculitis when its mass effect causes small bowel or lymphovascular obstruction (Duman et al., 2012).

#### Mesenteric fibrosis (MF)

As discussed, mesenteric panniculitis can develop sclerosing MF. Another condition that can cause MF is the jejunoileal neuroendocrine tumor (JI-NET) (Ahmed, 2020). Most of these tumors are located within 60 cm of the ileocecal valve. By the time diagnosis is made, they are >2 cm in size and have metastasized to the regional lymph nodes and liver (20% of cases) (Klöppel et al., 2004). JI-NETs can cause desmoplastic reactions leading to MF in 50% of patients (Druce et al., 2010). They secrete many profibrotic mediators, which include epidermal growth factor (EGF), fibroblast growth factor 2 (FGF2), fibroblast growth factor β (β-FGF), transforming growth factor beta and alpha (TGF-β and TGF-α), Insulin-like growth factor 1 (IGF-1), connective tissue growth factor (CTGF) and platelet-derived growth factor (PDGF) (Koumarianou et al., 2020). MF generally starts at the site of locoregional lymph node metastasis. Clinically, patients may remain asymptomatic or present with 1) abdominal pain due to obstruction of the small bowel or transverse colon as a result of fibrotic adhesion and mass effect in the root of the mesentery or small bowel ischemia due to encasement of superior mesenteric vein (SMV) or superior mesenteric artery (SMA), 2) abdominal distension as a consequence of transudative ascites owing to occlusion of SMV or chylous ascites due to lymphatic obstruction and 3) lower gastrointestinal bleed (GI) due to development of collaterals in the root of the mesentery or small bowel varices, and 4) malabsorptive diarrhea due to venous stasis (Ratnayake et al., 2022). Contrast-enhanced CT or multidetector CT can detect MF as an enhancing soft-tissue mass with linear soft tissue opacities/strands radiating outwards in the mesenteric fat in a “wheel spoke” or “stellate” pattern near a lymph node metastasis (Sundin et al., 2017). The severity of MF does not correlate well with the patient’s clinical symptoms or prognosis (Pantongrag-Brown et al., 1995). 68Ga-PET-CT–CT should also be done to identify the primary NET and its lymph node metastasis. Treatment is surgical resection of MF in symptomatic patients. Surgery is not advocated in asymptomatic patients with mesenteric fibrosis.

#### Mesenteric adiposity

Mesenteric fat is a continuous white adipose tissue (mWAT) attached around different parts of the intestine and is drained into the portal circulation. It is connected to the intestinal serosa and muscularis propria directly. It acts as a gate of communication between the intestine and other systems of the body. The sonographic average mesenteric fat thickness is <1 cm (range 0.22–1.7 cm). Males have greater mesenteric fat thickness than females. As per the “portal hypothesis,” excessive hepatic delivery of free fatty acid from mesenteric fat can lead to hepatic insulin resistance and metabolic derangement (Rytka et al., 2011). Mesenteric fat thickness ≥1 cm is an independent determinant of metabolic syndrome (sensitivity of 70% and specificity of 75%.) (Liu et al., 2006a), obesity, diabetes, accelerated atherosclerosis, cardiovascular diseases (Liu et al., 2003), obstructive sleep apnea (Liu et al., 2014), and metabolic associated steatotic liver disease (Liu et al., 2006b). Mesenteric adiposity is common in Crohn’s disease (CD) and plays a specific role in the pathogenesis of the disease (Peyrin-Biroulet et al., 2007). Mesenteric fat that migrates and wraps around the circumference of the inflamed small and large intestine in CD is called “creeping fat” and is pathognomonic of CD. This “creeping” migration of fat generally occurs when there is bacterial translocation from the transmurally inflamed intestine into mesenteric fat, and this happens in an attempt to prevent bacteria from spreading into the bloodstream. Creeping fat infiltrated by immune cells is immunologically active and secretes various pro-inflammatory (tumor necrosis factor-alpha (TNFα), interleukin-(IL)-6 (IL-6) and (IL-8) and anti-inflammatory cytokines and profibrotic factors, and is associated with the development of muscular propria hyperplasia and intestinal fibrosis (Aggeletopoulou et al., 2023). Creeping fat is a landmark for surgeons to localize the inflamed intestine in CD (Smith and Bénézech, 2020). Mesenteric adipocytes secrete C-reactive protein in response to local inflammation and bacterial translocation to mesenteric fat in Crohn’s disease (Peyrin-Biroulet et al., 2012). Increased mesenteric fat has been associated with increased Crohn’s disease activity, complications, decreased endoscopic healing, and increased post-surgical complications following ileal pouch anal anastomosis (Bilski et al., 2019).

Mesenteric cysts are rare benign cystic lesions in the mesentery with an incidence of one in 100,000 to 250,000 hospital admissions (Liew et al., 1994). They can be detected at any age with a slight female predominance. They are generally single but can be unilocular or multilocular with septations. Their size may vary from a few millimeters to a giant size (20–30 cm). They can be located anywhere in the mesentery, but most (60%) mesenteric cysts are seen in the mesentery proper (Pithawa et al., 2014). The cyst content is serous fluid or chyle. The exact etiopathogenesis of forming mesenteric cysts is unknown. It was proposed that ectopic lymphatics in the mesentery proliferate without any communication with the main lymphatics and form cysts (Richard, 2006). Other postulated mechanisms include trauma, degeneration of lymph nodes, and continued growth of congenitally malformed lymphatic tissue. Clinically, patients may remain asymptomatic, and the mesenteric cyst is detected in imaging studies during the workup of other conditions. Patients may have non-specific symptoms like abdominal pain, nausea, vomiting, or alteration of bowel habits. Patients may also present with abdominal mass or acute abdomen (Prakash et al., 2010). Acute abdomen generally occurs when mesenteric cysts develop complications, which include infection, intestinal obstruction, volvulus, torsion, rupture, bleeding, and hemorrhagic shock (Hardin and Hardy, 1970). Cross-sectional imaging by ultrasound, CT, and MRI (magnetic resonance imaging) should be done to evaluate the location and size of the cyst, the thickness of the wall, septations, content, and fluid level (Rajendran et al., 2014). Treatment is complete surgical excision of symptomatic mesenteric cysts by laparoscopic or open technique. The prognosis is good as the recurrence is low after surgical excision (Jain et al., 2012).

Mesenteric volvulus (MV) occurs due to twisting a loop of the intestine around its supporting mesentery and blood vessels. MV can lead to acute, subacute, or chronic strangulating bowel obstruction. Mesenteric volvulus can occur in the small intestine and colon. Small intestinal mesenteric volvulus (SIMV) generally occurs in infants, children, and young adults with small bowel malrotation. In adults, it is a rare cause of mechanical small intestinal obstruction. The incidence of SIMV is high (24–60 per 100,000 populations) in Asia, Africa, and Middle Eastern countries but low (1.7–5.7 per 100,000 populations) in Western countries (Awedew et al., 2020). Primary SIMV occurs due to segmental torsion of the small bowel at the base of the mesentery without any apparent cause. Secondary SIMV occurs due to adhesion, bands, stromal tumors, Meckel’s diverticulum, mesenteric hiatus hernia, and malrotation (Gürleyik and Gürleyik, 1998). The ileum is the most common site of SIMV. Patients generally present with abdominal pain, distension, nausea, vomiting, and obstipation. A plain X-ray of the abdomen shows few air-fluid levels with a lack of gas throughout the intestine but cannot detect specific signs of SIMV. CT scan should be done in suspected cases of SIMV. It may show a “whirl sign” – (a swirling of vessels in the mesenteric root) at the site of volvulus (Peterson et al., 2009). The diagnostic rate of detecting SIMV by CT scan is about 50% (Huang et al., 2005). As it is often impossible to diagnose SIMV before surgery, emergency surgery should be done to prevent small bowel necrosis and gangrene. The surgical intervention depends on the viability of the small bowel and the underlying cause of SIMV. Simple untwisting or devolvulation should be done if the small bowel is viable. In the case of gangrenous small bowel, resection of gangrenous segments followed by primary small bowel anastomosis should be done. The underlying causes, like adhesion, bands, internal hernia, stromal tumors, diverticulum, and malrotation, should also be treated surgically (Li et al., 2017). The incidence of colonic volvulus is low (<5%) in the Western world (North America, Western Europe, and Australia) but high (13%–42%) in the “volvulus belt” countries (South America, Eastern Europe, Middle East and South Asia) (Bhandari et al., 2019). In clinical practice, sigmoid volvulus (SV) is most commonly seen (60%–75%), followed by cecal volvulus (25%–40%). Rarely, volvulus can occur in the transverse colon (3%) and splenic flexure (2%). Colonic volvulus is a rare (1.90%) cause of colonic obstruction in the United States (Halabi et al., 2014). About 8% of all cases of intestinal obstruction are caused by SV. SV is more common in elderly males (median age 70 years), black race (because of their long sigmoid colon and narrow sigmoid mesocolon) (Madiba and Haffajee, 2011), adults with immobility, chronic constipation with chronic use of laxatives or enema, diabetes mellitus, neuropsychiatric disorders (Parkinson’s disease, Alzheimer’s disease, chronic schizophrenia, multiple sclerosis, Duchene muscular dystrophy, visceral myopathy), megacolon, neuroleptic medications and high fiber intake with overloading of sigmoid colon (Le et al., 2022a). Counterclockwise torsion of dilated stool-loaded sigmoid colon around its mesenteric axis forms sigmoid volvulus. Patients generally present with abdominal pain, distension, nausea, vomiting, and obstipation. Delay in diagnosis may lead to ischemic necrosis, hematochezia, perforation, and peritonitis.

A plain X-ray of the abdomen shows a markedly distended ahaustral sigmoid colon in the shape of a coffee bean (coffee bean sign or kidney bean sign) with a thick inner wall and a thin outer wall (bent inner tube sign). The loop classically points to the right upper quadrant. On the supine film, the apex of the sigmoid volvulus can be seen above the level of the transverse colon (northern exposure sign). Another diagnostic sign is the Frimann-Dahl sign, which shows three dense lines representing sigmoid colon walls converging to the site of torsion and absent rectal gas. Sometimes, the sigmoid volvulus can be seen ascending to the right upper quadrant and overlapping the liver shadow (liver overlap sign). CT findings of sigmoid volvulus include disproportionately enlarged ahaustral sigmoid colon with closed-loop obstruction. A whirl sign (twisting of mesentery and mesenteric vessels) can be present in about half of the cases. If rectal contrast is administered during CT, it may show gradual narrowing of the dilated sigmoid colon up to the level of obstruction (bird’s beak sign) (Jones et al., 2022). Management of sigmoid volvulus starts with stabilizing the patient by giving an infusion of fluid and electrolytes. A nasogastric tube should be placed. Colonoscopy is considered the first-line treatment to untwist and deflate the sigmoid volvulus in patients with uncomplicated sigmoid volvulus. After successful devolvulation, the patient immediately evacuates stool and gas *per rectum*, and the abdomen becomes soft. Emergent surgical intervention (sigmoidectomy with primary anastomosis or end colostomy) should be done if:

1. Colonoscopy is unsuccessful in correcting the volvulus.
2. Mucosa looks blackish/gangrenous during colonoscopy.
3. Signs of peritonitis on clinical examination and pneumoperitoneum on imaging.

Elective surgery should be considered in patients with recurrent attacks of sigmoid volvulus. Elective surgery has the advantage of having fewer post-operative complications, less open surgery, and less need for stoma (Lee et al., 2020). In patients with recurrent volvulus and poor surgical risk, successful colonoscopy-assisted percutaneous sigmoidopexy has been reported (Imakita et al., 2019).

Cecal volvulus (CV) occurs due to axial twisting or folding of a mobile cecum. Three types of CV have been observed (Le et al., 2022b):

Type 1 – occurs due to the clockwise twisting of the cecum along its long axis. CV is located in the right lower quadrant.

Type 2 occurs due to twisting a part of the cecum and a part of the terminal ileum in a counterclockwise direction (most of the time). CV is generally located in the left upper quadrant.

Type 3 (also called cecal bascule)– cecum is folded upwards without axial twisting.

About 1% of intestinal obstruction can be caused by CV. CV is more common in young females. From 2002 to 2010, the incidence of CV increased by 5.53% per year (Halabi et al., 2014). Patients present with abdominal pain, distention of the abdomen, nausea, and vomiting. A plain X-ray of the abdomen shows a distended cecum extending from the right lower quadrant to the left upper quadrant of the abdomen and a dilated small bowel with an air-fluid level. CT may show distal colonic decompression, >10 cm cecal distension, the cecal apex in the left upper quadrant, ileocecal twist, transition points, and “whirl sign” (loop of cecum looks spiraled with engorged mesenteric vessels and attenuated fatty mesentery) and dilated small bowel (Rosenblat et al., 2010).

Surgical intervention within 24–72 h after diagnosis is the treatment of choice. In patients with non-gangrenous cecum, right hemicolectomy with ileocolonic anastomosis is preferable. In patients with gangrenous cecum, right hemicolectomy with temporary ileostomy followed by a reversal of ileostomy on a later date should be done. In hemodynamically unstable patients with viable cecum, manual detorsion with cecopexy alone or cecopexy with tube cecostomy can be done (O’Mara et al., 1979). Delayed surgical intervention is associated with high mortality (>30%).

Mesenteric hematoma occurs due to bleeding from peripheral mesenteric vessels. It can be caused by abdominal trauma, surgical complications, rupture of mesenteric aneurysm, pancreatitis, anti-coagulation therapy, vasculitis, and collagen vascular diseases (Karam and Hajj, 1977; Skudder and Craver, 1984; De Brito et al., 2006; Hosaka et al., 2006; Meissnitzer et al., 2014; Hirano et al., 2018). 1%–5% of cases of blunt abdominal trauma can cause small bowel and mesenteric injury. When a mesenteric hematoma occurs without any apparent cause, it is labeled as spontaneous mesenteric hematoma (SMH). Rupture of mesenteric hematoma into the small bowel has been reported (Shikata et al., 2016). Patients may be asymptomatic, and the diagnosis is made on incidental imaging. Patients may feel abdominal pain and nausea. Imaging studies like contrast-enhanced CT or MRI can detect mesenteric hematoma. Conservative management is preferred if the patient remains asymptomatic or there is no sign of active bleeding. In the case of active bleeding, hemostasis can be obtained by selective angiography and emblotherapy (Basukala et al., 2022). Surgical intervention is required in case of a sizeable symptomatic hematoma, continued bleeding, complicated mesenteric hematoma, and diagnostic difficulty (Tanioka et al., 2020).

Mesenteric lymphangioma (ML) or Mesenteric lymphatic malformation (MLM) is a low-flow vascular malformation predominantly seen in the pediatric population, while adult cases are rare. Failure to communicate lymphatic sacs with the venous drainage system leads to the formation of MLM (Cupido and Low, 2015). Another theory suggests that sequestration of lymphatic tissue during embryologic development results in the formation of MLM. In adults, inflammation, trauma, surgery, and radiation can also cause sequestration of lymphatic tissue (Rieker et al., 2000). ML has three histological types: capillary, cystic, and cavernous. Most of the patients with MLM are asymptomatic and detected in imaging studies. As the size of MLM increases, patients develop abdominal pain. Physical examination may reveal abdominal distension and a palpable mass. Patients may also present with complications like small bowel obstruction (due to volvulus), infection, bleeding, and rupture (Thapa et al., 2022). Ultrasound or CT scan may show unilocular or multilocular cystic mass with variable septal thickness and enhanced wall in the mesentery. Radical resection of MLM, including segmental bowel resection, is the treatment of choice to prevent recurrence (Lim et al., 2018).

Intestinal malrotation is another mesenteropathy primarily seen in newborn infants and rarely in adults. Between 5 and 11 weeks of gestation, the midgut undergoes 270 degrees’ counterclockwise rotation around the superior mesenteric vessels. Partial or complete failure of this rotation leads to intestinal malrotation. As a result, the small intestine is located on the right side of the abdomen, the colon on the left side of the abdomen, and the cecum and the appendix in the upper abdomen because of the attachment of the cecum to the duodenum by Ladd’s bands. In the United States, intestinal malrotation occurs in one in 500 live births, and most cases are diagnosed by 1 year of age (Eccleston et al., 2016). Patients may remain completely asymptomatic forever. But they can become symptomatic at any age when the intestine becomes obstructed or twisted by Ladd’s bands. Although emesis is the most common symptom in infants, adults present with abdominal pain in the majority of cases and less often with nausea and vomiting (Nehra and Goldstein, 2011). Less commonly, patients may present with acute abdomen due to volvulus formation or internal herniation. The upper gastrointestinal (UGI) series is the most widely used imaging study in infants. In contrast, abdominal and pelvic CT with oral and intravenous contrast is the imaging study of choice in adults. UGI series reveals the duodenojejunal flexure/ligament of Trietz to be located on the right side of the abdomen with dilatation of various segments of the duodenum (corkscrew sign). CT abdomen shows the absence of the third part of the duodenum, non-visualization of the cecum in the right lower quadrant, small bowel in the right side and colon in the left side of the abdomen, right-left inversion of SMA and SMV, and sometimes “whirlpool” sign due to twisting of blood vessels around the mesenteric pedicle (Alani and Rentea, 2024). Surgical intervention is needed for all symptomatic patients with intestinal malrotation, irrespective of age. The treatment of choice is Ladd’s procedure which includes reduction of volvulus if present, untwisting of small bowel in a counterclockwise direction, positioning of the small intestine and colon in neutral position with or without fixation, lysis of Ladd’s band, division of bands causing obstruction, placing the cecum in the right paravertebral gutter, kocherization, and widening of the base of the mesentery proper and finally appendectomy (Seymour and Andersen, 2005). Currently, there is no consensus guideline for managing asymptomatic intestinal malrotation (Graziano et al., 2015).

Heterotrophic mesenteric ossification (HMO) is a rare condition characterized by calcification and reactive bone formation at the base of the mesentery (Tonino et al., 2005). Few cases have been described in the literature. It almost always occurs following abdominal surgery, abdominal blunt trauma, abdominal stab wounds, and abdominal gunshot injuries (Binesh et al., 2012). Very rarely, there may not be any prior history of abdominal surgery or abdominal injury (Bovo et al., 2004). The exact pathogenesis is unknown. It likely happens secondary to tissue injury with infiltration of inflammatory cells. Endogenous calcium is released from the mitochondria and endoplasmic reticulum. Phospholipase activity is increased in the alkaline milieu with the release of free fatty acids (FFA) from phospholipids and calcium bonds with FFA, leading to dystrophic calcification (Zulfiqar et al., 2020). Inflammatory cells (leucocytes, macrophages, and giant cells) activate mesenchymal cells to differentiate into osteoblasts and osteocytes (Fadare et al., 2002). Another hypothesis suggests that heterotopic ossification occurs due to “seeding” of the mesentery with activated osteoprogenitor cells during surgery or trauma (Mussato et al., 2016). Histologically, dense fibrous tissue, lamellar bone trabeculae, and mesenteric calcification are seen (Koolen et al., 2010). HMO is predominantly found in adult males. Patients can be completely asymptomatic or present with abdominal pain, nausea, vomiting, and abdominal distension due to small bowel obstruction (SB0). Diagnosis is made either by CT imaging or intra-operatively. As HMO is a benign condition, asymptomatic patients can be observed. Symptomatic patients should be treated with complete excision of mesenteric ossification.

## Primary mesenteric neoplasms

Primary mesenteric lymphoma (PML) is the most common malignant mesenteric neoplasm. It is a type of non-Hodgkin’s B cell lymphoma affecting mesenteric lymph nodes. Follicular lymphoma is more common than other histological types. It is extremely rare, with an incidence of about one in 200,000 to 350,000 population. Patients can be asymptomatic or present with anorexia, weight loss, abdominal pain, and abdominal mass (Ciortan and Carra, 2010). Rarely, patients may have autoimmune thrombocytopenia or dermatitis herpetiformis. PML can cause extrinsic compression on the bowel or encase mesenteric vessels. Cases of small bowel obstruction and gastrointestinal bleeding secondary to PML have been reported (Petkova et al., 2011; Kuroda and Lucia, 2022). CT or PET-CT scans may show mesenteric bulky homogenous soft tissue masses surrounding mesenteric vessels with intact perivascular fat space (“sandwich sign”). Diagnosis is established by ultrasound-guided or CT-guided biopsy of the mass. Treatment is combination chemotherapy.

Primary Mesenteric desmoid tumor (MDT) is the mesentery’s most common primary benign neoplasm. It is a rare fibromuscular neoplasm arising from mesenchymal cell lines. In the general population, the incidence is 2–4 per million per year (Dahn et al., 1963). The two essential characteristics of this tumor are 1) local aggressiveness causing mass effects or invasion into surrounding structures and 2) a high chance (30%–80%) of recurrence following excision (Daryani et al., 2008). It can occur sporadically or as an extra-intestinal manifestation of Familial adenomatous polyposis (FAP), Gardner’s or Turcot syndrome. 85%–90% of sporadic desmoid tumors are due to mutation of the gene CTNNB1, leading to increased intranuclear accumulation of β-catenin in these tumors. About 10%–15% of desmoid tumors are due to germline mutations in the APC (adenomatous polyposis coli) gene. As CTNNB1 and APC gene mutations are mutually exclusive, the finding of CTNNB1 gene mutation excludes APC gene mutation-related syndromes. Histologically, it shows characteristic proliferation of uniform spindle cells and myofibroblasts in long vesicles in the background of dense collagenous stroma and thin-walled blood vessels (Zreik and Fritchie, 2016). Females are more commonly affected than males, and the average age of presentation is 30–40 years. Patients can be asymptomatic when MDT is small, but as it increases in size, patients develop abdominal pain and abdominal mass, which can cause bowel or ureteral obstruction (D et al., 2014). Other complications include bowel perforation, intralesional abscess formation, and fistulization to adjacent bowel (Huang et al., 2017; Hajri et al., 2022). Cross-sectional imaging like CT or MRI (hypointense or isointense with respect to muscle on T1-weighted images and hyperintensity on T2-weighted images) shows a homogenous solid soft tissue mass most of the time in the mesentery displacing surrounding structures and vessels. Occasionally, heterogenous mass can be seen as well (Einstein et al., 1991). MRI is more sensitive in detecting the extent of the tumor and surrounding organ infiltration (Master et al., 2023). Tissue diagnosis is established by CT-guided percutaneous biopsy, EUS-guided fine needle aspiration, core needle biopsy, or laparoscopic surgical biopsy (Dalén et al., 2006). A pathologist with expertise in sarcoma or desmoid tumors should evaluate the tissue. In the case of resectable MDT, surgery with R0 resection is the treatment of choice if a negative margin can be obtained without functional impairment. But as MDT can invade surrounding organs, R0 excision may not be possible without resection of surrounding organs.

Another approach is to “watch and wait” if MDT does not cause any functional impairment. In case of a non-resectable tumor, incompletely resected tumor or recurrent MDT, radiotherapy or low-dose chemotherapy (methotrexate, vinblastine/vinorelbine, and doxorubicin-based regimen) or targeted therapy with tyrosine kinase inhibitors (imatinib, pazopanib, and sorafenib) should be offered to prevent progression of MDT (Ghert et al., 2014; Napolitano et al., 2020). A CT scan shows a solid mesenteric mass with or without necrosis.

Mesenteric leiomyosarcoma (MLS) is an extremely rare malignant tumor most likely arising from the smooth muscle of mesenteric blood vessels (Yannopoulos and Stout, 1963). The most common site of MLM is mesoileum, followed by transverse mesocolon and sigmoid mesocolon. Middle-aged individuals with female predominance are most commonly affected. Patients may remain asymptomatic or present with abdominal pain, abdominal distension, a palpable mass, or small bowel obstruction (Sharma et al., 2015). Percutaneous cutaneous biopsy is not recommended because of the risk of peritoneal and cutaneous dissemination of malignant cells. Diagnosis is confirmed by histopathology and immunohistochemistry (positive for desmin but negative for CD34, CD117, DOG1/delay of germination 1 and S-100) of the surgical specimen. The treatment of choice is surgical resection of the MLM with a wide margin of normal tissue (at least 4 inches) (Lee, 1983). MLM can recur within 5 years following resection. So, patients should be followed up closely for 5 years or more. The prognosis is poor, with a 5-year survival of 20%–30% (Fukunaga, 2004).

Primary mesenteric neuroendocrine tumors (NETs) arise from the neuroendocrine or the Kulchitsky cells in the mesentery. These are extremely rare tumors, and very few cases have been reported in the literature (Kamath et al., 2015). Most of the mesenteric NETs are due to local metastasis from the intestine. Patients with primary mesenteric NET may remain asymptomatic for an extended period and are detected incidentally in imaging studies. Patients develop symptoms with the increase in size of the tumor. Patients may present with abdominal pain, abdominal mass, small bowel obstruction, or carcinoid syndrome due to liver metastasis (Park et al., 2013). A CT scan may show a well-defined mass with a “spoke wheel” appearance (due to fibrosis, retraction, and calcification) and neurovascular bundle invasion (Karahan et al., 2006; Giambelluca et al., 2019). Serum chromogranin A is raised most of the time. Ga-68 PET (DOTATOC or DOTATATE) scan is a sensitive and specific imaging modality to detect the primary mesenteric NET and any metastasis. Surgical excision of the mesenteric NET is the treatment of choice. Diagnosis is confirmed by histopathology and immunohistochemistry (positive Synaptophysin, chromogranin A, cytokeratins, and neuron-specific enolase) (Bellizzi, 2020).

Primary mesenteric gastrointestinal stromal tumors (GISTs) are sporadic. They arise from the interstitial cells of Cajal (ICC) present in the mesentery. Most of the patients are above the age of 50 years. Patients may remain asymptomatic for a long time when the tumor size is small. Symptomatic patients generally present with abdominal pain, abdominal distension, or abdominal mass. Cross-sectional imaging (US, CT, MRI) shows a solid or solid and cystic mass arising from the mesentery (Ramani et al., 2017). The tissue sample can be obtained by ultrasound-guided or CT-guided biopsy. Diagnosis is confirmed by histopathology (70% spindle-cell type and 20% epithelioid type) and immunohistochemistry showing DOG1 stain positivity or overexpression of tyrosine kinase receptors as a result of mutation of C-kit genes (CD 117 or CD34) in 95% of cases or platelet-derived growth factor receptor A (PDGFRA) in 5% of cases (Puneet et al., 2009). The GIST carries an aggressive course if the size is >5 cm with a mitotic rate of >5/50 HPF. Treatment of primary mesenteric GIST is resection followed by TKI therapy. Preoperative TKI should be considered to decrease the size of the GIST if the patient has multiple comorbidities. TKI should be continued for an indefinite period. CT scan should be done: 1) every 6 months for 5 years for low-risk category patients, and 2) every 3 months for 3 years, then every 6 months for 5 years, and then yearly for intermediate to high-risk category patients (Casali et al., 2008). PET/CT is more sensitive than CT in detecting response, resistance, and recurrence following TKI therapy (Alberini et al., 2007).

## Summary

Although mesentery was considered a fragmented structure between the small and large intestines for many years, it was recognized as a single continuous organ in 2017. It is a membranous organ formed by a double fold of peritoneum and extends from the duodenojejunal flexure to the anorectal junction in a fan-like fashion. It has two layers separated by loose connective tissue and contains mesenteric blood vessels, lymphatic vessels, lymph nodes, nerves, and fat. Mesentery has multiple functions, which include mechanical function attaching the intestines to the posterior abdominal wall, lymphovascular communication between the intestine and the rest of the body, immune homeostasis, food allergy and tolerance, energy homeostasis by storing fat, and CRP secretion in response to local inflammation. Mesenteric defects, congenital or acquired, can cause internal hernias and intestinal obstruction. Mesenteric adenitis is an acute benign condition and responds well to hydration and NSAID. Mesenteric panniculitis is a chronic benign inflammatory condition of the mesenteric fat with characteristic imaging and histology. Patients can be asymptomatic or may present with abdominal pain. Treatment includes “watch and wait” for asymptomatic patients and anti-inflammatory medications for symptomatic medications. MF is due to JI-NET with mesenteric lymph node metastasis and desmoplastic reaction. The patient may be symptom-free or may present with abdominal pain, distension, GI bleeding, or diarrhea. A CT scan may show a “wheel spoke” appearance of soft tissue mass in the mesentery. Symptomatic patients need surgical resection of MF. Mesenteric adiposity is associated with metabolic syndrome, metabolic-associated steatotic liver disease, diabetes mellitus, cardiovascular diseases, and obstructive sleep apnea syndrome. Mesenteric cysts are a benign but rare condition. Patients can be asymptomatic with incidental detection on imaging studies or present with abdominal pain, nausea, or vomiting. Resection of the mesenteric cyst is the treatment of choice in symptomatic patients. Mesenteric volvulus can lead to intestinal obstruction with strangulation. CT of SIMV may show the “whirl sign” in 50% of cases. Emergency surgery should be done in suspected cases. Colonic volvulus is mainly seen in the “volvulus belt” countries but is rare in the Western world. Sigmoid volvulus is much more common than cecal volvulus. Patients present with abdominal pain and distension, and imaging studies confirm the diagnosis. In the case of uncomplicated sigmoid volvulus, colonoscopy is considered the first line treatment to devaluate, whereas surgery should be done as soon as possible in the case of cecal volvulus. Patients with mesenteric hematoma can be diagnosed as an incidental finding on imaging studies, or they may present with abdominal pain. Conservative treatment is preferred for asymptomatic patients. Emblotherapy or surgical intervention should be done for symptomatic patients or mesenteric hematomas with continued bleeding or complications. HMO is a rare condition that can be incidentally detected in imaging studies in asymptomatic patients. Patients with HMO may also present with abdominal pain or SBO. Asymptomatic patients can be observed, but surgical resection of HMO is required in the case of symptomatic patients. Primary mesenteric neoplasms are rare to sporadic. Imaging studies detect them, and diagnoses are confirmed by EUS-guided, CT-guided, or laparoscopic biopsy and sometimes surgical exploration of the abdomen. Surgical resection is the treatment of choice in most of these primary mesenteric neoplasms, except in the case of PML, where combination chemotherapy is the treatment of choice. The prognosis of benign non-vascular mesenteric lesions can be excellent if they can be treated early, either by medications or surgery.
